# Integrating multiple genome annotation databases improves the interpretation of microarray gene expression data

**DOI:** 10.1186/1471-2164-11-50

**Published:** 2010-01-20

**Authors:** Jun Yin, Sarah McLoughlin, Ian B Jeffery, Antonino Glaviano, Breandan Kennedy, Desmond G Higgins

**Affiliations:** 1 School of Medicine and Medical Science, Conway Institute, University College Dublin, Dublin, Ireland; 2 School of Biomolecular and Biomedical Science, Conway Institute, University College Dublin, Dublin, Ireland

## Abstract

**Background:**

The Affymetrix GeneChip is a widely used gene expression profiling platform. Since the chips were originally designed, the genome databases and gene definitions have been considerably updated. Thus, more accurate interpretation of microarray data requires parallel updating of the specificity of GeneChip probes. We propose a new probe remapping protocol, using the zebrafish GeneChips as an example, by removing nonspecific probes, and grouping the probes into transcript level probe sets using an integrated zebrafish genome annotation. This genome annotation is based on combining transcript information from multiple databases. This new remapping protocol, especially the new genome annotation, is shown here to be an important factor in improving the interpretation of gene expression microarray data.

**Results:**

Transcript data from the RefSeq, GenBank and Ensembl databases were downloaded from the UCSC genome browser, and integrated to generate a combined zebrafish genome annotation. Affymetrix probes were filtered and remapped according to the new annotation. The influence of transcript collection and gene definition methods was tested using two microarray data sets. Compared to remapping using a single database, this new remapping protocol results in up to 20% more probes being retained in the remapping, leading to approximately 1,000 more genes being detected. The differentially expressed gene lists are consequently increased by up to 30%. We are also able to detect up to three times more alternative splicing events. A small number of the bioinformatics predictions were confirmed using real-time PCR validation.

**Conclusions:**

By combining gene definitions from multiple databases, it is possible to greatly increase the numbers of genes and splice variants that can be detected in microarray gene expression experiments.

## Background

Microarrays are widely used to profile gene expression patterns in samples of biological material. Affymetrix GeneChips are a popular oligonucleotide microarray platform, using probe sets formed by 11-20 pairs of 25 mer probes. The probe pairs include a perfect match probe (PM) and a single base mismatch (MM) probe targeting the gene transcripts. These probes were originally selected from the consensus sequence alignments of expressed sequence tag (EST) sequences. Over the past 10 years, the sequences and annotations of the main genomes have changed significantly. One consequence has been that some of the EST sequences were re-annotated or even removed from the databases. Thus, probes targeting these ESTs are no longer accurate [1]. This problem has created a need to remap the probes using information from the most up to date genome sequence databases. Since the GeneChips were introduced, the importance of alternative splicing, especially in vertebrates, has become more and more apparent. The annotation of these transcripts has changed considerably over recent years and this has also increased the importance of using the latest and most comprehensive genome annotation databases to map probes to specific transcripts.

Several probe-remapping protocols have been developed, generally by regrouping the probes to the target genes or transcripts according to the current version of genome annotation [1-5]. A crucial consideration in probe remapping is the annotation database usage. Dai et al. provided several probe remappings, each using a different database, e.g. Unigene, RefSeq and Ensembl [1]. However, this may lead to difficulty and confusion when interpreting microarray results. The problems come from i) genes annotated in one database but not in the other databases; ii) genes having longer transcripts or more transcripts in one database, but shorter or smaller in another. This usually means that the expression level or alternative splicing events of the gene can only be detected using one database but not using the other. The remapping results from Lu et al., Lee et al., and Moll et al. either used the Refseq and Aceview databases, or the Ensembl database [1-3,5]. The results may be very different if the database is changed.

RefSeq and Ensembl are two widely used genome annotation databases [6,7]. Though the data are regularly transferred between these databases, incompatible transcripts or genes are discarded during the transfer thus leading to discrepancies. Aceview provides comprehensive genome annotation by integrating data from RefSeq, dbEST and GenBank [8]. However, only five species were annotated in Aceview, which means that Aceview annotation cannot be easily used in other species. ZFIN is a highly accurate, manually corrected zebrafish genome annotation database, integrating RefSeq and GenBank transcripts [9]. Its strict criteria, however, may result in the loss of a certain amount of transcript data. Furthermore, ZFIN does not provide cross-referenced transcript information to Ensembl transcript data. These variations in the genome annotation may lead to difficulties in interpreting gene expression results. Thus, there is a need for a comprehensive and unbiased genome annotation. The UCSC genome browser provides a comprehensive genome annotation for more than 40 species [10]. The data from the UCSC genome browser can be easily accessed and used to provide customized genome annotations.

A well established remapping method is used in AffyProbeMiner and several other protocols [2-5], which regroup the probes into a probe set if they all match the same set of transcripts. This transcript level probe remapping provides the possibility to detect alternatively spliced transcripts. However, it does not provide an appropriate method to measure the levels of alternative splicing events. Mainly due to the recent development of exon arrays, algorithms predicting alternative splicing have been developed (see [11] for a recent review). The exon array algorithms should be carefully used for 3' gene expression microarray data, however. The oligo-dT based amplification method used in 3' gene expression microarray has a strong position effect rendering a signal bias towards the probes targeting the 3' ends of genes. A normalized intensity based method, such as Splicing Index [12,13], is more appropriate to avoid this signal bias.

Here we report a new probe remapping protocol and demonstrate its use with the zebrafish genome. It is based on a combined zebrafish genome annotation by integrating transcripts from the Ensembl, RefSeq and GenBank databases using information downloaded from the UCSC genome browser. A transcript level probe remapping is applied by aligning the probes to the genome, removing the nonspecific probes, and grouping the probes according to the set of transcripts they map to. We explore the impact of using different databases for gene and transcript annotations. We also used the Splicing Index [12,13] as an indicator of alternative splicing events. The advantage of using a comprehensive database in the probe remapping was demonstrated as more genes and more alternative splicing events are detected. Using two different zebrafish gene expression experiments, we show the benefits of using the more comprehensive remapping and confirm the improvement using real-time PCR validation.

## Results

### Probe remapping of the Zebrafish Genome Array

**Figure 1:**
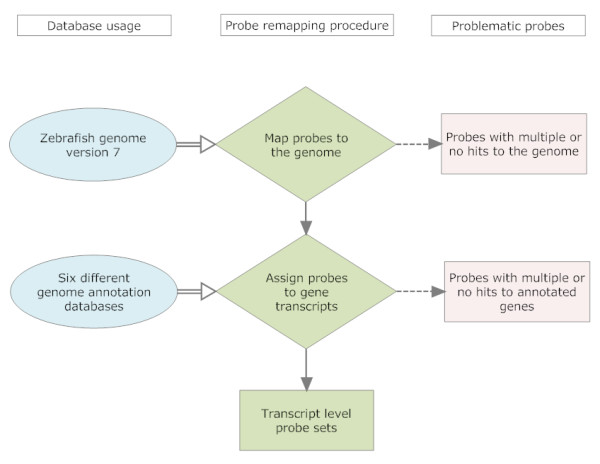
**Work flow showing an outline of the probe remapping procedure**.

The probe remapping procedure is outlined in Figure 1. Firstly problematic probes were removed. This included probes with genome location issues where probes have no match to the current genome or have multiple matches. It also included probes with unique matches to the genome but which match multiple genes. Then the probes were regrouped into new probe sets according to the set of transcripts which they match. This step is clearly, highly dependent on the annotation database that is used and this is the major focus of this paper. We illustrate this process in detail with the zebrafish genome and a commonly used gene expression platform from Affymetrix.

### Mapping probes to the genome

To identify probes that have genome location issues, we aligned all 249,752 probes from the Affymetrix Zebrafish Genome Array to the genome of the zebrafish (genome version 7 (Zv7)) using Exonerate [14]. 19,585 of the probes were identified with multiple matches and about 40,000 probes were identified with no match to the current genome. Further details and analysis of probes matching multiple genome locations is provided in Additional File 1. Thus, about 24% of probes were nonspecific for the genome. By removing these problematic probes, we are left with about 190,000 genome specific probes. The number of probes having genome location issues is strikingly high. The reason is mainly because Affymetrix originally designed the probes based on Expressed Sequence Tags (ESTs) from a number of databases e.g. Unigene, GenBank, dbEST [15]. Some of these EST sequences were erroneous and their removal from updated databases results in the loss of the probes. We regard the removal of probes having genome location issues as an acceptable loss of signal in order to avoid erroneous mapping of the probes to unannotated exons or genes.

### Alignment of transcripts to the genome

In order to group the probes into transcript level probe sets, we identify those genome positions corresponding to coding regions. This is done by aligning transcripts from different databases onto the genome. We used six databases that define zebrafish genes based on collections of transcripts. These are are the single source databases: GenBank, RefSeq, Ensembl, and three multiple source databases: Biomart, ZFIN and UCSC. The latter three integrate transcripts from the three single source databases. These databases differ in their collections of transcripts. Transcripts which did not properly align to the genome are usually due to mistakes in earlier versions of the genome assembly and/or EST collections. For example in GenBank, 59,527 transcripts are included in the database, while only 25,336 can be aligned to the current genome. Importantly, in our remapping, Affymetrix probes originally designed from erroneous transcripts are removed. As demonstrated in Table 1, databases that use multiple sources encompass more transcripts. The UCSC genome browser has the largest collection of zebrafish transcripts with 75,723 alignable transcripts, while RefSeq only has 13,172 transcripts alignable to the current genome. We organized transcripts from the UCSC genome browser into 28,110 genes by clustering transcripts overlapping in coding exons. Genes defined in other databases were according to cross reference files as describe in the *Methods *section.

**Table 1:** Number of transcripts and genes from each database and number of alignable genes and transcripts in the UCSC genome browser

Database	No. of transcripts	No. of genes	No. of alignable transcripts	No. of alignable genes
**Single data source**				
GenBank	59,527	36,843	25,336	14,130
RefSeq	30,499	28,999	13,304	13,172
Ensembl	35,967	25,546	35,967	25,546
**Multiple data sources**^**1**^				
ZFIN	79,424	21,430	52,246	14,217
Biomart	61,047	21,322	58,135	21,322
UCSC	75,723	28,110^2^	75,723	28,110

^1^: These databases integrate transcripts from Ensembl, RefSeq and GenBank databases.

^2^: Genes are defined by clustering transcripts overlapping in coding exons

### Assignment of probes to gene specific transcript level probe sets

About 190,000 genome specific probes were mapped to gene transcripts annotated from the 6 different databases. We explored the impact of database usage on the number of probes retained during probe remapping (Table 2). The number of probes with no gene annotations is strongly affected by the gene annotation database that is used. These contain probes which matched either intergenic regions or gene introns. The removal of these probes results in huge differences among databases, ranging from 65,443 to 91,090 in total. Other problematic probes accounted for a minor percentage of all the probes (<3.6%). This includes probes matching multiple genes, and probes that fail to meet the minimum criterion of 3 probes to form a probe set. Multiple source databases usually included more probes by providing a more comprehensive annotation of the genome. Approximately 47.2% of the probes were retained after probe remapping using the UCSC database, which was the largest percentage. Only 38.1% of probes were retained using Ensembl.

**Table 2:** Summary of probe remapping results using different databases

	GenBank	RefSeq	Ensembl	ZFIN	Biomart	UCSC
Probes with multiple alignments to the genome	19,585	19,585	19,585	19,585	19,585	19,585
Probes with no alignment to the genome	41,609	42,398	41,972	41,613	41,488	41,077
Probes matching multiple genes	2,718	1,674	432	2,228	1,592	1,487
Probes matching intergenic region	65,220	75,916	81,273	64,394	66,806	54,532
Probes matching intron region	8,348	6,912	9,817	9,049	10,068	10,911
< 3 probes per probe set	2,369	762	1,464	3,884	3,126	4,378
Good probes	109,903	102,505	95,209	108,999	107,087	117,782
Percentage of good probes	44.005%	41.043%	38.121%	43.643%	42.877%	47.160%
Probe sets	8,574	6,769	7,156	9,195	8,732	10,251

Probe remapping using the UCSC database allowed the highest number of probes to be retained, mainly because it integrates the GenBank database. The Affymetrix Zebrafish Genome Array was originally designed from GenBank [15]. This shows the necessity of integrating GenBank in the probe remappings, which has been neglected by some probe remapping protocols [1,16].

### Genes annotated by remapped probe sets using different databases

Using multiple source databases results in retaining more probes and therefore, more transcripts and genes representative of the genome. Using UCSC, 7725 genes are detected. This is about 27% of the genes in the zebrafish genome (Table 3). 2,069 genes were represented by more than 2 probe sets. With these genes we also have the possibility to measure alternative splicing patterns. The average number of probe sets per gene indicates the ability to detect alternative splicing events using the remapped probe sets. The largest number was obtained using UCSC with 1.327 probe sets per gene. In contrast, using Ensembl, only 6,347 genes are covered by the probes, with 1.127 probe sets per gene.

**Table 3:** Summary of genes and transcripts matched by the probes using different databases

	**GenBank**	**RefSeq**	**Ensembl**	**ZFIN**	**Biomart**	**UCSC**
	
Number of transcripts matched by the probes	12,803	6,688	8,076	25,665	23,212	28,356
Number of genes matched by the probes	7,069	6,560	6,347	7,003	6,983	7,725
Number of genes matched by ≥ 2 probe sets	1,296	202	726	1,809	1,439	2,069
Average number of transcripts per probe set	1.493	0.988	1.129	2.791	2.658	2.766
Average number of probe sets per gene	1.213	1.032	1.127	1.313	1.250	1.327

### Pairwise comparison of probe remapping using different databases

Differences in the probe remapping results will affect the interpretation of results from microarray data analysis. This can be seen in lists of differentially expressed genes. For genes to have the same probe annotation using different databases we require the probes targeting this gene to be the same and to be clustered into identical probe sets. Genes have different probe annotations using different databases, either because genes in a subset of databases have more probes in the probe set, or the original probe set is split into separate probe sets to represent alternative splicing transcripts.

We compared the gene probe sets obtained using the three single source databases to the probe remapping using UCSC (Table 4). Only half of the genes were mapped by the same probe sets using different databases. The largest agreement was between GenBank and UCSC with 5,442 genes sharing the same probe set content. The number of genes having a probe set annotation in UCSC but not in the other databases is quite large, accounting for about 1,000 genes. These genes can only be detected using UCSC gene annotation. Large differences between databases may lead to the difficulties in interpreting microarray data.

**Table 4:** Comparison of genes represented by the remapped probe sets using different databases

Database A	Database B	Same	Diff	UniqueA	UniqueB
UCSC	Ensembl	3,979	2,329	1,417	60
UCSC	RefSeq	3,551	2,955	1,219	406
UCSC	GenBank	5,442	1,627	656	637

Same: number of genes having the same probe sets in A and B.

Diff: number of genes having different probe sets in A and B.

UniqueA: number of genes having probe sets in A but not in B.

UniqueB: number of genes having probe sets in B but not in A.

### Impact of gene definitions on probe remapping

The differences between the gene annotation databases are due to the different transcript collections and gene definition methods used. Gene definition methods generally decide how different transcripts are clustered into genes. In order to investigate how gene definition alone affects microarray data analysis results, four different gene definition methods were applied to the transcript collection from UCSC and the probe remapping results were compared. Gene transcript clustering is either performed by comparing transcript intron/exon boundary locations (*itbd *and *exbd*) or overlap region in the exon sequences (*exlink *and *overlap_0*). *Exlink *is the default gene definition method used here, which clusters transcripts overlapping in coding exons. The other methods are compared with it (Table 5, Table 6). The largest difference was identified with *exbd*. *Exbd *is the most stringent definition which requires both exon boundaries of at least one exon to match. It makes *exbd *an outlier in the number of genes defined and number of probes retained after remapping. Although the number of probes retained using *Exbd *can be up to 3% fewer than with *exlink*, the genes represented by the remapped probe sets were very similar. More than 91% of genes were represented by the same probe sets between *exlink *and *exbd*. Thus gene definition methods contribute only a small amount to the differences in probe remapping results and microarray data interpretation.

**Table 5:** Comparison of genes represented by the remapped probe sets using different gene definitions

Database A	Database B	Same	Diff	UniqueA	UniqueB
*exlink*	*exbd*	7,044	215	466	21
*exlink*	*itbd*	7,448	157	120	64
*exlink*	*overlap_0*	7,659	66	0	111

Same: number of genes having the same probe sets in A and B.

Diff: number of genes having different probe sets in A and B.

UniqueA: number of genes having probe sets in A but not in B.

UniqueB: number of genes having probe sets in B but not in A.

**Table 6:** Summary of gene definition and probe remapping results using different gene definition methods

	***exlink***	***exbd***	***itbd***	***overlap_0***
	
**Gene definition**				
Number of transcripts in the database	75,723	75,723	75,723	75,723
Number of genes defined by the method	28,110	32,686	29,501	27,876
**Probe remapping result**				
Probes having genome location issues	60,662	60,662	60,662	60,662
Probes matching multiple genes	1,487	10,672	4,360	974
Probes matching no gene	65,443	65,443	65,443	65,443
< 3 probes per probe set	4,378	3,992	4,234	4,402
Good Probes	117,782	108,983	115,053	118,271
Percentage of Good Probes	47.160%	43.636%	46.067%	47.355%

### Impact of probe remapping on two biological data sets

**Figure 2:**
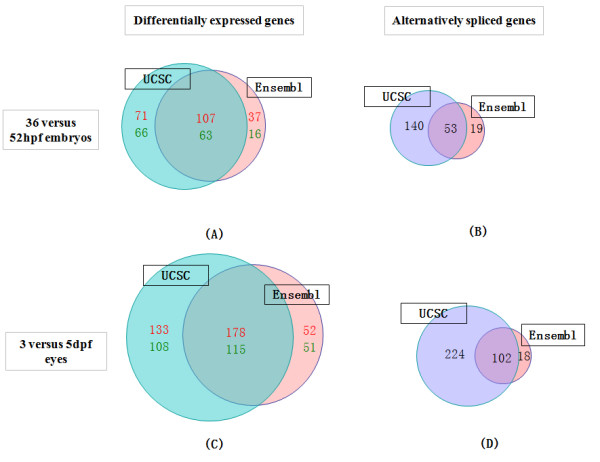
**Comparison of gene lists generated by remapped probe sets using the UCSC and Ensembl databases**. A, C: number of differentially expressed genes. Number of up-regulated genes is in red. Number of down-regulated genes is in green. B, D: number of alternative spliced genes. A, B: 36 versus 52 hpf whole zebrafish embryos data set. C, D: 3 versus 5 dpf zebrafish eyes data set

We examined how database usage influences the interpretation of gene expression data from real microarray experiment data. Two biological data sets were used. First we analyzed a published gene expression dataset [17] comparing whole zebrafish embryos at 36 and 52 hours post fertilisation (hpf). A second, in-house, data set analysed gene expression in zebrafish eyes at 3 and 5 days post fertilisation (dpf). Probe remapping using the single source database, Ensembl, and the multiple source database, UCSC, were applied. Microarray data analysis was performed using these two customized probe definitions. The eBayes t-test and Splicing Index were used to select differentially expressed genes, and genes showing alternative splicing patterns, respectively, as describe in the *Methods *section. Probe remapping using these two databases generated significantly different gene lists (Figure 2).

The differentially expressed genes that were identified using Ensembl were mostly included in the gene list generated using UCSC. This shows that probe remapping using UCSC gives more extensive gene lists when searching for differentially expressed genes. A further benefit of using the multiple source database, UCSC, is the ability to predict more alternative splicing events. Exclusively more genes were identified showing alternative splicing patterns using UCSC than using Ensembl.

**Figure 3:**
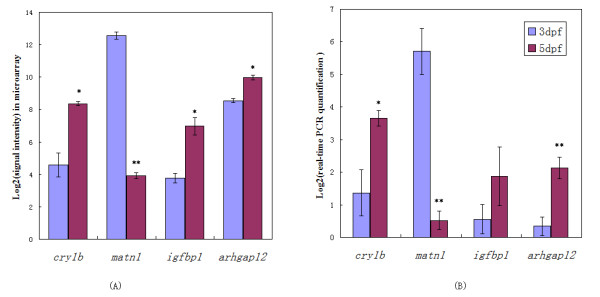
**Real-time PCR validation of differentially expressed genes in the 3 versus 5 dpf zebrafish eyes data set**. These genes can only be detected by remapping using the multiple source database, UCSC. (A) Signal intensities from microarray data. (B) Real-time PCR results depicted as relative abundance compared to lowest abundance sample. *: p-value < 0.05. **: p-value < 0.01.

A few genes which were interpreted differently using these two databases in the 3 versus 5 dpf eyes gene lists were selected for experimental validation using real-time PCR. Four genes show significant differential expression in the 3 versus 5 dpf eyes, but are only detected using the multiple source database, UCSC (Figure 3, Additional file 2). The real-time PCR results prove that all these transcripts are expressed and not an artefact of our analysis. Three out of the four genes were validated as significantly differentially expressed by real-time PCR analyses.

**Figure 4:**
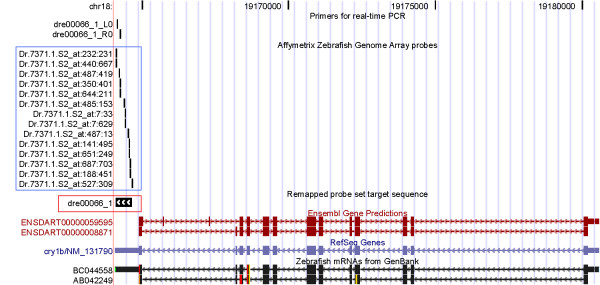
**Schematic view of *cry1b *showing the advantage of integrating multiple databases**. Dr.7371.1.S2_at is the original Affymetrix probe set targeting *cry1b *as shown in the blue square. dre00066_1 is the remapped probe set as shown in red square. Affymetrix probes match the RefSeq transcript NM_131790 and the GenBank transcript BC044558but no Ensembl transcript is matched by this probe set. dre00066_1_L0 and dre00066_1_R0 are primers used in the real-time PCR.

The gene *cry1b *is illustrated as an example in Figure 4. *Cry1b *encodes the cryptochrome 1b protein which is reported to regulate circadian rhythms [18]. Five transcripts were annotated for *cry1b *gene in the UCSC genome browser, two from Ensembl, one from RefSeq and two from GenBank. Affymetrix probes only targeted two *cry1b *transcripts, NM_131790 and BC044558, from RefSeq and GenBank respectively. The *cry1b *gene was identified in the microarray as having -3.8 log2 fold change (fold change of 0.078, qvalue = 0.026) and verified using real-time PCR as -2.3 log2 fold change (fold change of 0.204, pvalue = 0.019).

**Figure 5:**
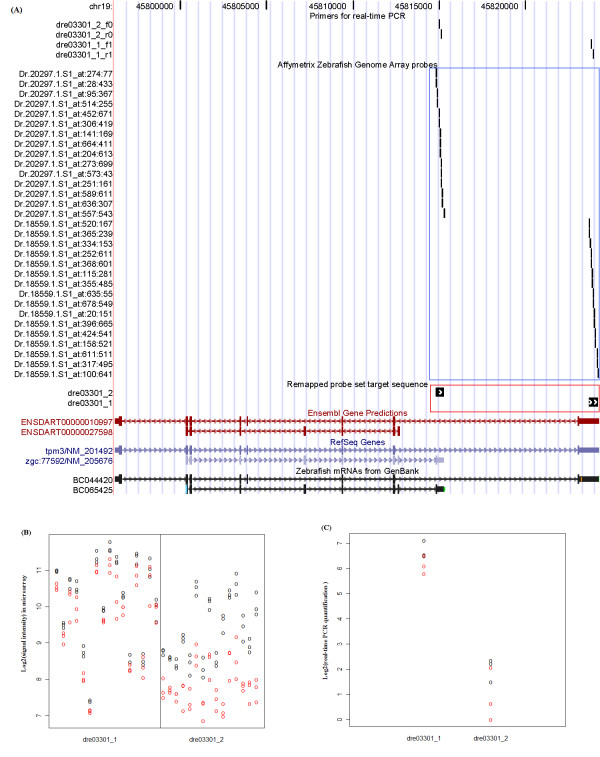
**Schematic view of *tpm3 *showing the advantage of integrating multiple databases in revealing alternative splicing pattern**. (A) Schematic view of *tpm3*. dre03301_1 and dre03301_2 (in the red square) are remapped probe sets with probes from Dr.18559.1.S1_at and Dr.20297.1.S1_at (in the blue square) respectively. (B) Log-base 2 signals of the probes from the remapped probe sets with 3dpf and 5dpf gene expression in black and red dots, respectively. (C) The real-time PCR results depicted as relative quantification compared to lowest abundance sample.

We use *tpm3 *as an example for the verification of alternative splicing (Figure 5). *Tpm3 *encodes a tropomyosin family actin-binding protein involved in muscle contraction [19]. One of the probe sets for *tpm3*, dre03301_1, can only be mapped using the RefSeq and GenBank databases. The log2 fold changes for the *tpm3 *probe sets in the microarray results were 0.540 and 4.941 (fold changes of 1.456 and 31.565 respectively), and verified as 0.582 and 1.118 (fold changes of 1.497 and 2.171 respectively) in the real-time PCR results. The real-time PCR result confirmed that the *tpm3 *transcripts annotated in RefSeq and GenBank are expressed, and are true splice variants of the transcript annotated in Ensembl.

## Discussion

### Gene specific transcript level probe remapping protocol

Several protocols have been published to improve probe remapping of Affymetrix microarrays. The general protocol is to remove the problematic probes and group the remainder by the gene or transcript that they target. The protocol that we implement here has been optimised to use a combination of features from existing protocols [1,3-5]. Casneuf et al. reported that expression of nonspecific probes was highly correlated with off-target genes [16]. Thus, we removed the genome-nonspecific probes in order to minimize the off-target probe pairing. This is more stringent than previous published methods. AffyProbeMiner has provided transcript consistent and gene consistent probe sets by clustering probes matching the same set of transcripts and genes [4]. However, probes matching multiple genes should be avoided. Moll et al. aligned the probes to the transcriptome [1,2]. Yet this may include probes that match unannotated transcripts, or transcripts which are non-alignable to the current genome.

How to group the probes into probe sets is a further concern in probe remapping. The transcript level probe remapping should reveal differences in gene splice isoforms. Dai et al. provided transcript targeted probe sets by grouping probes targeting individual transcripts [1] but this generated redundant probes in the remapping. The transcript level probe remapping used in this study is more appropriate as it clusters probes when they match the same set of transcripts [2-4]. Moll et al. applied a similar method and validated the splice variants by real time PCR [2]. Lu et al. also reported that this transcript level remapping reduced the platform variance between microarrays [3]. None of them, however, provided any method to measure the expression variation of gene splice isoforms.

Li et al. developed an ANOVA model based method to calculate the variance of the sibling Affymetrix probe sets [20]. This model is widely used with Affymetrix exon microarrays to detect splice isoform variation [11]. However, 3' gene expression microarrays have a strong signal bias towards probes targeting the 3' ends of genes. The probe hybridization efficiency with targeting genes may also affect the signal strength. These signal biases may affect the ANOVA model by giving false positive p-values. The Splicing Index calculates the tissue specific expression by pair-wise comparison of the normalized intensities [12,13] and therefore, is more appropriate for 3' gene expression microarrays. Thus, in this work, we used the Splicing Index to measure the alternative splicing patterns in 3' gene expression microarrays.

### Impact of database usage on microarray analysis

Database usage is a major concern in microarray data analysis. As shown by Dai et al., the difference in probe set content using different databases caused a 30-50% difference in differentially expressed gene lists [1]. Moll et al. compared their remapping results for HG-U133A with the AffyProbeMiner mapping [2]. AffyProbeMiner defined 10,226 probe sets using RefSeq, whereas Moll et al. yielded 7,941 probe sets using Ensembl. Only 3,412 probe sets were identical between the two approaches. Our study reported a similar result. The number of genes having the same probe sets across databases ranged from 3,551 to 5,442 when compared between single source databases and the multiple source database UCSC (Table 4). However, none of the previous protocols provided any appropriate method to reconcile the large differences between the databases. Dai et al. provided downloadable remapping results using each individual database [1]. AffyProbeMiner reported that using RefSeq and GenBank together may improve the mapping of the probes [4]. Unfortunately, the database integration method used by AffyProbeMiner was computationally intensive using BLAT alignments of GenBank transcripts to the genome sequences. Furthermore, they did not integrate transcript information from Ensembl.

Here, we provide a more practical genome annotation method by downloading transcript information from UCSC, and clustering the transcripts by overlapping coding exons (*exlink*). This protocol was implemented in a Perl script and the genome reannotation can be finished within minutes. This protocol can be applied to any of the more than 40 species deposited in the UCSC genome browser to rebuild the genome annotation [10]. *Exlink *provides a biologically meaningful annotation, and can easily be applied to all species with published genome sequences. It should be pointed out, however, that the gene definition methods compared in this manuscript, *exbd*, *itbd *and *exlink*, from RefSeq, Aceview and Ensembl respectively, all involve manual correction. Thus it is impossible to fully repeat their work in our study.

The issues described here become even more serious during the analysis of data from next-generation RNA sequencing [21]. In these analyses, 20-25% of the good quality reads with unique matches to the genome cannot be mapped to annotated genes in Ensembl or Eldorado. It suggests our knowledge of the genomes is still limited and much work still needs to be done to improve the genome annotation. The annotation provided in this study is a combination of the information from three databases. This is easily applied and is essential in fully interpreting such large-scale data sets.

## Conclusions

We developed an improved probe remapping protocol based on mapping probes to the genome sequence, removing nonspecific probes and grouping the probes into transcript level probe sets. The protocol is based on a combined zebrafish genome annotation by integrating the Ensembl, RefSeq and GenBank databases together. This integrated genome annotation will reduce database variation bias in large scale gene expression studies. The data analysis protocol used in this study improves the interpretation of gene expression data. This approach could easily be applied to other species and gene expression measurement platforms such as exon microarrays or RNA seq.

## Methods

### Data sources

Zebrafish transcriptome cross reference files were downloaded from Ensembl (Zv7, July 2007), RefSeq (Zv7 Build3, July 2008), GenBank (October 23, 2008), Biomart (Zv7, Ensembl 52 genes) and ZFIN (October 23, 2008) (Table 1). The cross-reference files link the transcript IDs with gene IDs. Zebrafish genome sequences, transcriptome alignment coordinates and coding sequence (CDS) coordinates were downloaded from the UCSC genome browser. Only transcripts with 96% base identity with the genomic sequence were kept. If the transcripts had multiple alignments to the genome, only alignments having a base identity level within 0.1% of the best for RefSeq transcripts, and 0.5% of the best for GenBank transcripts were kept [10]. The Affymetrix Zebrafish Genome Array probe sequences and Chip Description File (CDF) were downloaded from NetAffx (October 23, 2008) [15].

### Probe mapping and redefinition of the probe sets

The remapping protocol was adapted from a probe remapping protocol described by Dai in 2005 [1]. The remapping was performed as follows. The Affymetrix probe sequences were aligned to the zebrafish genome using Exonerate [14]. Only probes that had perfect sequence identity with the genome were used in the study. Probes with no match to the genome or which matched multiple times, were removed, because these probes may match unannotated genes. These two filters ensure the probes will hybridize to a specific location in the genome. An exception is that probes having no match to the genome, but which match transcript sequences, were considered as probes which cross exon boundaries, and were included in this analysis . This was performed by assembling transcript sequences from the GenBank, RefSeq and Ensembl databases from the transcribed regions of the genome. The Affymetrix probe sequences were aligned to the transcript sequences using Exonerate [14]. .

Probes which matched multiple genes were removed because these probes may generate nonspecific signals. This was due to probes mapping to the overlapping untranslated regions (UTRs) of pairs of genes. Probes matched to the intergenic regions or introns of genes were removed. Reverse complementary probes were organized into a different probe set. Because these probes targeted the opposite strand of the transcript, they usually generate a much weaker signal than the probes targeting the positive strand of the transcript (further analysis of this is given in Additional File 1).

The major change from Dai's protocol was that probes were reorganized in transcript-level probe sets by clustering probes matching the same set of transcripts, in order to measure transcript level expression. Apart from the above filters, we also required that each new probe set should include more than 3 probes (see Additional File 1 for further details). The remapped probe set definition was transformed into new probe sequence and CDF packages using the Bioconductor packages, *matchprobes *[22] and *makecdfenv *[23]. R libraries of the probe and CDF packages, and annotation of the remapped probe sets are provided in Additional File 3. The probe remapping protocol is implemented in a Perl script, and provided in Additional File 4.

### Gene definition

*Exlink*, the gene definition proposed by Ensembl was used in the study in order to organize transcripts from multiple databases [6]. Transcripts overlapping in coding exons were clustered in the same gene. Gene annotation using *exlink *is provided as Additional File 5. Several other gene definitions were also used to demonstrate the impact of gene definition on the probe remapping. *Itbd*, the gene definition proposed by Aceview [8], clusters all transcripts which share at least one intron boundary. *Exbd*, the gene definition proposed by RefSeq, clusters all transcripts sharing both boundaries of at least one exon [24]. *Overlap_0*, the old gene definition used by Ensembl, clusters all transcripts which overlap in the exon sequences [25]. The gene definition protocols are implemented in Perl scripts, provided in Additional File 4.

### Microarray experiment

Eyes were dissected from 3 and 5 days post fertilization (dpf) zebrafish larvae, and total RNA extracted with Qiashredder columns and the RNeasy Minikit (Qiagen, Hilden, Germany) in an RNase-free environment. RNA was quantified using the Nanodrop ND-1000 (ThermoScientific) and quality was determined using RNA 6000 Pico chips with the Bioanalyzer 2100 (Agilent). Three biological replicates per timepoint with equal amounts of RNA were amplified and labelled using a two-cycle target labelling protocol (Affymetrix) and hybridised with Affymetrix Zebrafish Genome Arrays. The 3 and 5 dpf eyes microarray data set was deposited in GEO with series accession ID of GSE19320. A published microarray data set studying 36 and 52 hours post fertilization (hpf) whole zebrafish embryos was downloaded from GEO, with sample accession IDs from GSM224790 to GSM224796 [17]. All experimental research on animals followed internationally recognized guidelines and approval from the UCD Animal Research Ethics Committee.

### Microarray data analysis

The signal intensity of the microarray was normalized and summarized using the Bioconductor package, *gcrma *[26]. Differentially expressed genes were selected by using Bioconductor package, *limma *[27]. The eBayes p-value was adjusted by using Benjamini & Hochberg's method [28]. The threshold for differentially expressed genes was set as adjusted p-value < 0.05 and fold change ≥2 or ≤0.5.

### Predicting splice variants

For genes with multiple probe sets, the Splicing Index [12,13] is calculated in order to predict tissue specific alternative splicing patterns. First, the signal of each probe set for the gene is normalized. The normalized intensity for probe set i in tissue x (NI_i, x_) is calculated as the signal intensity of probe set i in tissue x (P_i, x_) divided by the signal intensity of gene G in tissue x (G_x_).(1)

After obtaining the tissue specific normalized intensity for the probe set, the expression of the probe set among different tissues can be compared. When only two tissue specific expressions are measured in the experiment, SI is calculated as:(2)

Where NI_i,1 _is the normalized intensity of the probe set i in the first tissue, and NI_i,2 _in the second tissue.

If the Splicing Index for any probe set of a gene is ≥0.5 or ≤-0.5, this gene is predicted to be alternatively spliced [29]. The probe set expressions are used separately to indicate transcript level expression. If the Splicing Indexes for all probe sets in this gene are below this threshold, the probe set expressions can be averaged to indicate the gene level expression.

### Real-time PCR validation

To validate the microarray results, real-time PCR was used. Total RNA was extracted from zebrafish eyes as described above. Three biological replicates per time-point with equal amounts of RNA were reverse transcribed to cDNA with random hexamers using the SuperScript III First-Strand Synthesis System (Invitrogen, UK). Negative controls were synthesized using the same reaction without SuperScript III enzyme. Real-time PCR was performed on three biological replicates per timepoint using the ABI 7900HT Sequence Detection System with SYBR Green as the reporter. The initial cycle was 2 minutes at 50°C and 10 minutes at 95°C. Then the samples were cycled at 95°C for 15 seconds and 60°C for 1 minute. 18s rRNA primers were used as control. The primers were designed using Primer3 [30] and synthesised by Eurofins MWG Operon (Germany). Primer sequences are listed in Additional File 2. All primers showed specific amplification in real-time PCR. Water and negative controls were not detected in the real-time PCR (Ct>40). Real-time data were normalized according to 18s rRNA, and standardized to the lowest abundance value. The algorithm was illustrated using Microsoft Excel in Additional File 2.

## Authors' contributions

The project was conceived by JY and supervised by BK and DH. All data analysis was carried out by JY with advice and input from IBJ and DH. The RNA extraction and PCR were done by JY under the guidance of SMcL and BK. The microarray gene expression experiments were done by AG under the guidance of BK. JY wrote the manuscript with input from all authors. All authors read and approved the final manuscript.

## Supplementary Material

Additional file 1 Further investigation of parameters used in probe remapping Click here for file

Additional file 2 Real-time PCR primer sequences and results Click here for file

Additional file 3 Probe sequence and CDF R libraries of probe remapping using UCSC database, and annotation of the remapped probe sets Click here for file

Additional file 4 Perl scripts mapping probes, and clustering gene transcripts Click here for file

Additional file 5 Gene annotation of UCSC transcripts by clustering transcripts overlapping in coding exons Click here for file

## References

[B1] Dai M Wang P Boyd AD Kostov G Athey B Jones EG Bunney WE Myers RM Speed TP Akil H Watson SJ Meng F Evolving gene/transcript definitions significantly alter the interpretation of GeneChip data Nucleic Acids Res 2005 33 e175 10.1093/nar/gni179 16284200 PMC1283542

[B2] Moll AG Lindenmeyer MT Kretzler M Nelson PJ Zimmer R Cohen CD Transcript-specific expression profiles derived from sequence-based analysis of standard microarrays PLoS ONE 2009 4 e4702 10.1371/journal.pone.0004702 19277110 PMC2650090

[B3] Lu J Lee JC Salit ML Cam MC Transcript-based redefinition of grouped oligonucleotide probe sets using AceView: high-resolution annotation for microarrays BMC Bioinformatics 2007 8 108 10.1186/1471-2105-8-108 17394657 PMC1853115

[B4] Liu H Zeeberg BR Qu G Koru AG Ferrucci A Kahn A Ryan MC Nuhanovic A Munson PJ Reinhold WC Kane DW Weinstein JN AffyProbeMiner: a web resource for computing or retrieving accurately redefined Affymetrix probe sets Bioinformatics 2007 23 2385 2390 10.1093/bioinformatics/btm360 17660211

[B5] Lee JC Stiles D Lu J Cam MC A detailed transcript-level probe annotation reveals alternative splicing based microarray platform differences BMC Genomics 2007 8 284 10.1186/1471-2164-8-284 17708771 PMC2000902

[B6] Flicek P Aken BL Beal K Ballester B Caccamo M Chen Y Clarke L Coates G Cunningham F Cutts T Down T Dyer SC Eyre T Fitzgerald S Fernandez-Banet J Graf S Haider S Hammond M Holland R Howe KL Howe K Johnson N Jenkinson A Kahari A Keefe D Kokocinski F Kulesha E Lawson D Longden I Megy K Ensembl 2008 Nucleic Acids Res 2008 36 D707 714 10.1093/nar/gkm988 18000006 PMC2238821

[B7] Pruitt KD Tatusova T Maglott DR NCBI reference sequences (RefSeq): a curated non-redundant sequence database of genomes, transcripts and proteins Nucleic Acids Res 2007 35 D61 65 10.1093/nar/gkl842 17130148 PMC1716718

[B8] Thierry-Mieg D Thierry-Mieg J AceView: a comprehensive cDNA-supported gene and transcripts annotation Genome Biol 2006 7 Suppl 1 11 14 10.1186/gb-2006-7-s1-s12 PMC1810549 16925834

[B9] Sprague J Bayraktaroglu L Bradford Y Conlin T Dunn N Fashena D Frazer K Haendel M Howe DG Knight J Mani P Moxon SA Pich C Ramachandran S Schaper K Segerdell E Shao X Singer A Song P Sprunger B Van Slyke CE Westerfield M The Zebrafish Information Network: the zebrafish model organism database provides expanded support for genotypes and phenotypes Nucleic Acids Res 2008 36 D768 772 10.1093/nar/gkm956 17991680 PMC2238839

[B10] Karolchik D Kuhn RM Baertsch R Barber GP Clawson H Diekhans M Giardine B Harte RA Hinrichs AS Hsu F Kober KM Miller W Pedersen JS Pohl A Raney BJ Rhead B Rosenbloom KR Smith KE Stanke M Thakkapallayil A Trumbower H Wang T Zweig AS Haussler D Kent WJ The UCSC Genome Browser Database: 2008 update Nucleic Acids Res 2008 36 D773 779 10.1093/nar/gkm966 18086701 PMC2238835

[B11] Cuperlovic-Culf M Belacel N Culf AS Ouellette RJ Data analysis of alternative splicing microarrays Drug Discov Today 2006 11 983 990 10.1016/j.drudis.2006.09.011 17055407

[B12] Srinivasan K Shiue L Hayes JD Centers R Fitzwater S Loewen R Edmondson LR Bryant J Smith M Rommelfanger C Welch V Clark TA Sugnet CW Howe KJ Mandel-Gutfreund Y Ares M Jr Detection and measurement of alternative splicing using splicing-sensitive microarrays Methods 2005 37 345 359 10.1016/j.ymeth.2005.09.007 16314264

[B13] Clark TA Sugnet CW Ares M Jr Genomewide analysis of mRNA processing in yeast using splicing-specific microarrays Science 2002 296 907 910 10.1126/science.1069415 11988574

[B14] Slater GS Birney E Automated generation of heuristics for biological sequence comparison BMC Bioinformatics 2005 6 31 10.1186/1471-2105-6-31 15713233 PMC553969

[B15] Liu G Loraine AE Shigeta R Cline M Cheng J Valmeekam V Sun S Kulp D Siani-Rose MA NetAffx: Affymetrix probesets and annotations Nucleic Acids Res 2003 31 82 86 10.1093/nar/gkg121 12519953 PMC165568

[B16] Casneuf T Peer Y Van de Huber W In situ analysis of cross-hybridisation on microarrays and the inference of expression correlation BMC Bioinformatics 2007 8 461 10.1186/1471-2105-8-461 18039370 PMC2213692

[B17] Leung YF Ma P Link BA Dowling JE Factorial microarray analysis of zebrafish retinal development Proc Natl AcadSci USA 2008 105 12909 12914 10.1073/pnas.0806038105 PMC2529063 18753621

[B18] Kobayashi Y Ishikawa T Hirayama J Daiyasu H Kanai S Toh H Fukuda I Tsujimura T Terada N Kamei Y Yuba S Iwai S Todo T Molecular analysis of zebrafish photolyase/cryptochrome family: two types of cryptochromes present in zebrafish Genes Cells 2000 5 725 738 10.1046/j.1365-2443.2000.00364.x 10971654

[B19] Lees-Miller JP Helfman DM The molecular basis for tropomyosin isoform diversity Bioessays 1991 13 429 437 10.1002/bies.950130902 1796905

[B20] Li H Zhu D Cook M A statistical framework for consolidating "sibling" probe sets for Affymetrix GeneChip data BMC Genomics 2008 9 188 10.1186/1471-2164-9-188 18435860 PMC2397416

[B21] Sultan M Schulz MH Richard H Magen A Klingenhoff A Scherf M Seifert M Borodina T Soldatov A Parkhomchuk D Schmidt D O'Keeffe S Haas S Vingron M Lehrach H Yaspo ML A global view of gene activity and alternative splicing by deep sequencing of the human transcriptome Science 2008 321 956 960 10.1126/science.1160342 18599741

[B22] Huber W Gentleman R matchprobes: a Bioconductor package for the sequence-matching of microarray probe elements Bioinformatics 2004 20 1651 1652 10.1093/bioinformatics/bth133 14988118

[B23] Irizarry RA Gautier L Huber W Bolstad BM makecdfenv: CDF Environment Maker R package version 1160 2006 http://www.bioconductor.org/packages/2.2/bioc/html/makecdfenv.html

[B24] The NCBI Handbook 2003 http://www.ncbi.nlm.nih.gov/bookshelf/br.fcgi?book=handbook

[B25] Curwen V Eyras E Andrews TD Clarke L Mongin E Searle SM Clamp M The Ensembl automatic gene annotation system Genome Res 2004 14 942 950 10.1101/gr.1858004 15123590 PMC479124

[B26] Wu Z Irizarry R Gentleman R Murillo FM Spencer F A Model-Based Background Adjustment for Oligonucleotide Expression Arrays Journal of the American Statistical Association 2004 99 909 917 10.1198/016214504000000683

[B27] Smyth GK Linear models and empirical bayes methods for assessing differential expression in microarray experiments Stat Appl Genet Mol Biol 2004 3 Article3 16646809 10.2202/1544-6115.1027

[B28] Benjamini Y Hochberg Y Controlling the false discovery rate: a practical and powerful approach to multiple testing Journal of the Royal Statistical Society 1995 57 289 300

[B29] Affymetrix Identifying and Validating Alternative Splicing Events Technical Note http://www.affymetrix.com/support/technical/technotes/id_altsplicingevents_technote.pdf

[B30] Rozen S Skaletsky H Primer3 on the WWW for general users and for biologist programmers Methods Mol Biol 2000 132 365 386 10547847 10.1385/1-59259-192-2:365

