# Supplementary material for: Integrating multiple genome annotation databases improves the interpretation of microarray gene expression data
Source: BMC Genomics. 2010 Jan 20;11:50. doi: 10.1186/1471-2164-11-50 (PMC2827411; doi:10.1186/1471-2164-11-50)
Supplement: Additional file 3 — Probe sequence and CDF R libraries of probe remapping using UCSC database, and annotation of the remapped probe sets [file 1471-2164-11-50-S3.ZIP › dreucscjyincdf_1.16.0.zip!/dreucscjyincdf/html/geometry.html]

R: Convert (x,y)-coordinates to single-number indices and back.

|  |  |
| --- | --- |
| i2xy {dreucscjyincdf} | R Documentation |

## Convert (x,y)-coordinates to single-number indices and back.

### Description

Convert (x,y)-coordinates on the chip (and in the CEL file) to the
single-number indices used in AffyBatch and CDF environment, and back.

### Usage

```
i2xy(i)
xy2i(x,y)
```

### Arguments

|  |  |
| --- | --- |
| `x` | numeric. x-coordinate (from 1 to 712) |
| `y` | numeric. y-coordinate (from 1 to 712) |
| `i` | numeric. single-number index (from 1 to 506944) |

### Details

Type `i2xy` and `xy2i` at the **R** prompt to
view the function definitions.

### See Also

`dreucscjyincdf`

### Examples

```
  xy2i(5,5)
  i     = 1:(712*712)
  coord = i2xy(i)
  j     = xy2i(coord[, "x"], coord[, "y"])
  stopifnot(all(i==j))
  range(coord[, "x"])
  range(coord[, "y"])
```

---

[Package *dreucscjyincdf* version 1.16.0 Index]
