# Supplementary material for: Integrating multiple genome annotation databases improves the interpretation of microarray gene expression data
Source: BMC Genomics. 2010 Jan 20;11:50. doi: 10.1186/1471-2164-11-50 (PMC2827411; doi:10.1186/1471-2164-11-50)
Supplement: Additional file 3 — Probe sequence and CDF R libraries of probe remapping using UCSC database, and annotation of the remapped probe sets [file 1471-2164-11-50-S3.ZIP › dreucscjyinprobe_1.0.0.zip!/dreucscjyinprobe/html/00Index.html]

R: Probe sequence data for microarrays of type dreucscjyin

# Probe sequence data for microarrays of type dreucscjyin

---

## Documentation for package ‘dreucscjyinprobe’ version 1.0.0

## Help Pages

|  |  |
| --- | --- |
| dreucscjyinprobe | Probe sequence for microarrays of type dreucscjyin. |
