# Supplementary material for: Integrating multiple genome annotation databases improves the interpretation of microarray gene expression data
Source: BMC Genomics. 2010 Jan 20;11:50. doi: 10.1186/1471-2164-11-50 (PMC2827411; doi:10.1186/1471-2164-11-50)
Supplement: Additional file 3 — Probe sequence and CDF R libraries of probe remapping using UCSC database, and annotation of the remapped probe sets [file 1471-2164-11-50-S3.ZIP › dreucscjyinprobe_1.0.0.zip!/dreucscjyinprobe/html/dreucscjyinprobe.html]

R: Probe sequence for microarrays of type dreucscjyin.

|  |  |
| --- | --- |
| dreucscjyinprobe {dreucscjyinprobe} | R Documentation |

## Probe sequence for microarrays of type dreucscjyin.

### Description

This data object was automatically created by the package matchprobes version 1.10.0.

### Usage

```
data(dreucscjyinprobe)
```

### Format

A data frame with 119909 rows and 6 columns, as follows.

|  |  |  |
| --- | --- | --- |
| sequence | character | probe sequence |
| x | integer | x-coordinate on the array |
| y | integer | y-coordinate on the array |
| Probe.Set.Name | character | Affymetrix Probe Set Name |
| Probe.Interrogation.Position | integer | Probe Interrogation Position |
| Target.Strandedness | factor | Target Strandedness |

### Source

The probe sequence data was obtained from http://www.affymetrix.com.

### Examples

```
  dreucscjyinprobe
  as.data.frame(dreucscjyinprobe[1:3,])
```

---

[Package *dreucscjyinprobe* version 1.0.0 Index]
